# EEPD1: Breaking and Rescuing the Replication Fork

**DOI:** 10.1371/journal.pgen.1005742

**Published:** 2016-02-04

**Authors:** Yilun Liu

**Affiliations:** Department of Cancer Genetics and Epigenetics, Beckman Research Institute, City of Hope, Duarte, California, United States of America; University of Washington School of Medicine, UNITED STATES

**Fig 1:**
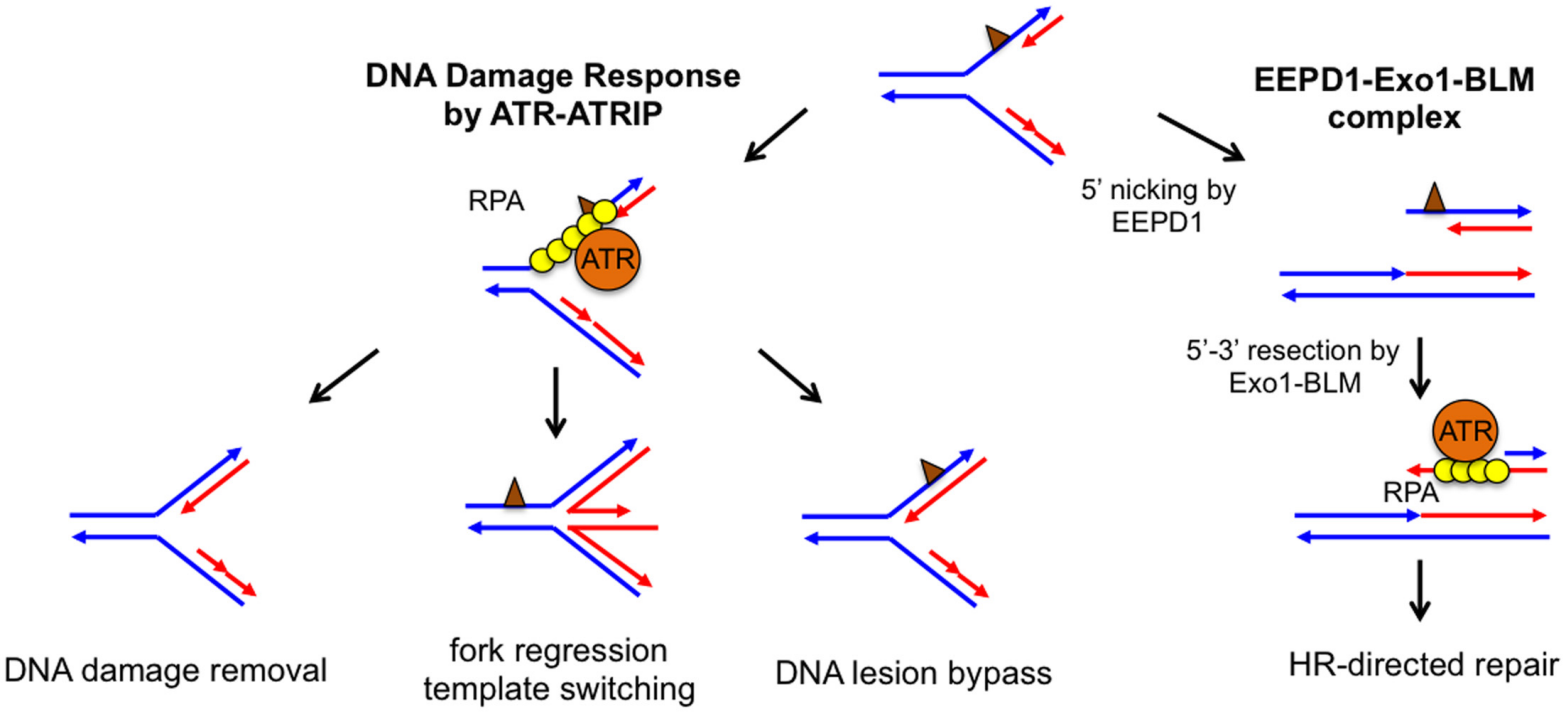
Potential pathways to repair and restart a stalled replication fork. Parental DNA strands (blue) and newly synthesized DNA strands (red) form a stalled fork due to the presence of DNA damage or replication blockage (brown triangle). Left: In the classical model, ssDNA at a stalled replication fork is bound by replication protein A (RPA), which recruits ataxia telangiectasia and Rad3-related protein (ATR) to activate the DNA damage response. ATR-dependent DNA damage response stabilizes the stalled replication fork, whose damage or blockage is repaired by DNA lesion bypass, direct damage removal, or template-switching via fork regression. Right: The EEPD1-Exo1-BLM) constitutive complex may provide an alternative pathway by nicking the 5′ single-stranded region of the stalled strand to generate a DNA double-stranded break, which is resected to form a 3′ single-stranded overhang to initiate homologous recombination (HR).

The faithful duplication of an entire genome is a complex affair requiring the coordinated action of the DNA replisome to unwind and synthesize DNA at replication forks. Unfortunately, exposure to chemicals or radiation can damage DNA strands, and this damage can stall DNA replication forks, resulting in genome instability, tumorigenesis, or cell death. To rescue stalled replication forks, cells have evolved a comprehensive network of DNA repair pathways that allow them to recover from these impediments (Fig 1) [1,2]. For example, either the direct chemical reversal of a damaged DNA base or DNA translesion synthesis allows a stalled replication fork to resume replication without disassembling the replisome. Alternatively, replication fork regression can generate a chicken foot structure, which provides the stalled strand with an undamaged sister chromatid to use as a template for DNA synthesis. This allows the replisome to bypass the damaged site. Fork regression also provides the cell an opportunity to repair DNA damage before converting chicken foot structures back to replication forks. However, because chicken foot structures resemble Holliday junctions (HJ), prolonged or unregulated fork regression may lead to DNA cleavage by HJ nucleases and DNA double-stranded breaks (DSBs).

Collapsed replication forks are frequently associated with DSB formation. Even though DSBs can be repaired by either homologous recombination (HR) or non-homologous end joining (NHEJ), DSBs are often the source of increased genomic instability [3,4]. For this reason, the recent discovery by Hromas, Nickoloff, and collaborators of a novel nuclease involved in DSB formation at stalled replication forks is quite interesting [5]. This team initiated their search for enzymes that are important for DNA damage repair by screening for genes induced by the topoisomerase IIα poison VP-16. They found that one of the up-regulated genes encodes a previously uncharacterized human protein named Exonuclease/Endonuclease/Phosphatase Domain-1 (EEPD1). The absence of EEPD1 slowed the rate of replication fork progression after hydroxyurea (HU) treatment, indicating that EEPD1 is important for replication fork recovery from replication stress. In addition, HR frequency was significantly reduced in EEPD1-deficient cells, suggesting that EEPD1 may have a role in repairing stalled replication forks by HR. If this is the case, one would predict that the decrease in HR efficiency would lead to the accumulation of unrepaired DSBs. Surprisingly, just the opposite occurred: DSBs decreased in EEPD1 deficient cells, as indicated by reduced γH2AX focus formation and the amount of tail moment detected by an alkaline comet assay. Therefore, EEPD1 is required for generating DSBs in response to replication stress. A regressed replication fork containing an HJ-like chicken foot structure could be nicked on two opposite strands by an HJ resolvase to create a DSB [1,2]. Even though EEPD1 contains a DNA binding domain similar to that found in the bacterial HJ binding protein RuvA, in vitro EEPD1 does not cleave HJ like an HJ resolvase [5]. Instead, EEPD1 exhibits a 5′ endonuclease activity similar to DNA2 nuclease. However, the depletion of DNA2 nuclease led to an increase in DSB formation instead of the decrease found in the EEPD1-depleted cells. This is due to the fact that in vivo DNA2 processes the regressed replication forks to promote replication fork recovery and prevent replication fork collapse [6]. Therefore, even though EEPD1 and DNA2 both exhibit 5′ nuclease activities in vitro, EEPD1 does not share the same function as DNA2 in repairing stalled replication forks. Most likely, EEPD1 nicks the 5′ single-stranded DNA (ssDNA) region of the replication fork to generate a DSB (Fig 1). DSBs at collapsed replication forks are thought be an undesirable byproduct, because DSBs may be repaired by error-prone NHEJ, which generates mutations and translocations leading to genomic instability [4]. What then could be the benefit of EEPD1-initiated DSB repair? Yuehan Wu and colleagues found that the DSBs produced by EEPD1 are repaired by HR, because EEPD1 forms a constitutive complex with Bloom’s syndrome helicase (BLM) and Exo1, whose 5′–3′ exonuclease activity efficiently resects the DSB ends to generate 3′ ssDNA overhangs necessary for the initiation of HR [5]. As a bonus, these overhangs cannot be repaired by error-prone NHEJ [7].

How does EEPD1-initiated DNA repair fit in the complex network of replication fork repair pathways? One might expect that the EEPD1 pathway functions as a failsafe mechanism, as this pathway causes the replication fork to collapse and leads to DSB formation. However, an isolation of protein on nascent DNA (iPOND) analysis showed that EEPD1 was enriched at the replication fork within 30 minutes of HU treatment, and EEPD1 depletion led to a decrease in replication protein A (RPA) foci and phosphorylation of ataxia telangiectasia and Rad3-related protein (ATR)/Chk1 after DNA damage [5]. ATR and ATR-interacting protein (ATRIP) are first responders that are recruited to a stalled replication fork by RPA-coated ssDNA to initiate the DNA damage response (DDR; Fig 1), and one of the important roles of the ATR-dependent DDR is to prevent replication fork collapse [1,2]. Could EEPD1 initiate DSB formation early in the replication stress response, thereby bypassing ATR-dependent DDR? If this is the case, which factor(s) may recruit EEPD1 to the stalled replication fork? Is it possible that EEPD1 is constitutively associated with normal replication forks, but is only activated when replisome progression slows due to DNA damage? Indeed, the same iPOND experiment also showed that EEPD1 is associated with the replication fork, even without the treatment with HU [5]. Whatever the answers, EEPD1 is clearly responsible for the activation of some ATR-dependent DDR. This raises the possibility that EEPD1-induced RPA foci and ATR activation are a response to DSB formation and the ssDNA overhangs generated by end resection. If so, one would predict that cells expressing an EEPD1 nuclease defective mutant would also exhibit reduced RPA foci and ATR activation.

In summary, this study provides compelling evidence that the EEPD1-dependent repair pathway for stalled replication forks is an early pathway choice that maintains genome stability in response to replication stress, and it raises fascinating questions for future research.
